# Functional similarity between TGF-beta type 2 and type 1 receptors in the female reproductive tract

**DOI:** 10.1038/s41598-021-88673-y

**Published:** 2021-04-29

**Authors:** Nan Ni, Xin Fang, Qinglei Li

**Affiliations:** Department of Veterinary Integrative Biosciences, Texas A&M University, College Station, TX 77843 USA

**Keywords:** Physiology, Reproductive biology

## Abstract

Transforming growth factor β (TGFβ) signaling plays critical roles in reproductive development and function. TGFβ ligands signal through the TGFβ receptor type 2 (TGFBR2)/TGFBR1 complex. As TGFBR2 and TGFBR1 form a signaling complex upon ligand stimulation, they are expected to be equally important for propagating TGFβ signaling that elicits cellular responses. However, several genetic studies challenge this concept and indicate that disruption of TGFBR2 or TGFBR1 may lead to contrasting phenotypic outcomes. We have shown that conditional deletion of *Tgfbr1* using anti-Mullerian hormone receptor type 2 (*Amhr2*)-Cre causes oviductal and myometrial defects. To determine the functional requirement of TGFBR2 in the female reproductive tract and the potential phenotypic divergence/similarity resulting from conditional ablation of either receptor, we generated mice harboring *Tgfbr2* deletion using the same Cre driver that was previously employed to target *Tgfbr1*. Herein, we found that conditional deletion of *Tgfbr2* led to a similar phenotype to that of *Tgfbr1* deletion in the female reproductive tract. Furthermore, genetic removal of *Tgfbr1* in the *Tgfbr2*-deleted uterus had minimal impact on the phenotype of *Tgfbr2* conditional knockout mice. In summary, our results reveal the functional similarity between TGFBR2 and TGFBR1 in maintaining the structural integrity of the female reproductive tract.

## Introduction

Major components of the transforming growth factor β (TGFβ) signaling include ligands, receptors, and SMAD proteins^1^. Binding of TGFβ ligands with their type 2 receptor (TGFBR2) induces the formation of TGFBR2/TGFBR1 complex, where TGFBR2 phosphorylates the Gly-Ser (GS) domain of TGFBR1^2^. TGFBR1 activation leads to subsequent phosphorylation of intracellular SMAD2/3, which cooperate with SMAD4 to regulate gene expression^1^. TGFβ superfamily proteins regulate multiple developmental processes and reproductive events including, but not limited to, embryonic development, organogenesis, and uterine development and function^3,4^. TGFBR1 is known to maintain the developmental integrity of the female reproductive tract^5^. We have previously shown that conditional deletion of *Tgfbr1* using anti-Mullerian hormone receptor type 2 (*Amhr2*)-Cre causes the formation of oviductal diverticula, myometrial defects, and endometrial hyperplasia^5–7^.

Existing literature has depicted the spatiotemporal expression of TGFBR2 in several reproductive events, usually in conjunction with TGFBR1 and/or TGFβ ligands. *Tgfbr2* mRNA is weakly expressed in the rat endometrium during estrous cycle^8^. However, *Tgfbr2* is expressed on pregnant days 0.5–1.5 and the expression is decreased on days 2.5–3.5^8^. Dynamic expression of *Tgfbr2* and *Tgfbr1* transcripts has been found during the peri-implantation period^8^. In the mouse, TGFBR2 and TGFBR1 are expressed in the oviduct on days 1–4 of pregnancy^9^. In the human, myometrial TGFBR2 is upregulated before the onset of parturition and then declined during spontaneous laboring^10^. TGFBR2 is also expressed in the placenta^11^. Additionally, TGFBR2 is aberrantly expressed in pathological conditions such as endometrial cancer and polycystic ovary syndrome^12,13^. These data suggest the involvement of TGFBR2-mediated signaling in the regulation of uterine function. However, the functional requirement for TGFBR2 in the female reproductive tract development has not been established.

As TGFBR1 functions to maintain the integrity of the female reproductive tract^5^ and TGFBR2 and TGFBR1 form a complex upon TGFβ signaling activation, it is conceivable that TGFBR2 and TGFBR1 are equally important in safeguarding the developmental integrity of the female reproductive tract. Supporting that TGFBR2 and TGFBR1 play a similar role, it was found that missense mutations in either *Tgfbr1* (M318R) or *Tgfbr2* (G357W), mutations associated with severe Loeys-Dietz syndrome in human patients, cause enhanced growth of the aortic root and enlarged aortas in mice^14^. However, elegant genetic studies in the vascular system, neural crest cells, and cartilage demonstrate that ablation of TGFBR2 or TGFBR1 promotes divergent phenotypes^15–18^. For instance, Yang and colleagues found that conditional deletion of *Tgfbr1* in vascular smooth muscle cells using myosin heavy chain 11 (*Myh11*)-Cre leads to severe aneurysmal degeneration in mice^15^. In contrast, conditional deletion of *Tgfbr2* results in milder pathological changes^15^. In addition, disruption of *Tgfbr2* mitigates the aortic pathology induced by *Tgfbr1* deletion, accompanied by attenuated ERK1/2 signaling^15^. It was proposed that abnormal TGFBR2 signaling induced by *Tgfbr1* deletion partially led to the aortic pathology^15^. Zhao and colleagues reported that conditional deletion of *Tgfbr1* in neural crest tissues causes delayed tooth initiation and impaired mandible patterning, phenotypes that are absent in *Tgfbr2* mutant mice^16^. Similarly, conditional depletion of TGFBR1 in the cartilage results in lethal chondrodysplasia, which is absent in *Tgfbr2* mutant mice^17^. Moreover, contrasting facial phenotypes between *Tgfbr2* and *Tgfbr1* mutants have been observed^18^. These studies indicate a contextually-dependent signaling divergence of TGFBR1 and TGFBR2. Therefore, it is critical to assess whether TGFBR2 and TGFBR1 function similarly in a given experimental system to understand their contextual interactions and provide rational basis for studies aimed at deciphering the role of TGFβ signaling via targeting either *Tgfbr2* or *Tgfbr1*, with an assumption that absence of either receptor similarly impairs the TGFβ signaling.

To determine the functional requirement of TGFBR2 in the female reproductive tract and clarify potential signaling divergence between TGFBR1 and TGFBR2, we conditionally deleted *Tgfbr2* using *Amhr2*-Cre and compared phenotypes between mice with targeted deletion of *Tgfbr2* and *Tgfbr1*. Mice with simultaneous deletion of *Tgfbr2* and *Tgfbr1* were also generated to substantiate the findings.

## Results

### Loss of TGFBR2 disrupts myometrial formation

**Figure 1:**
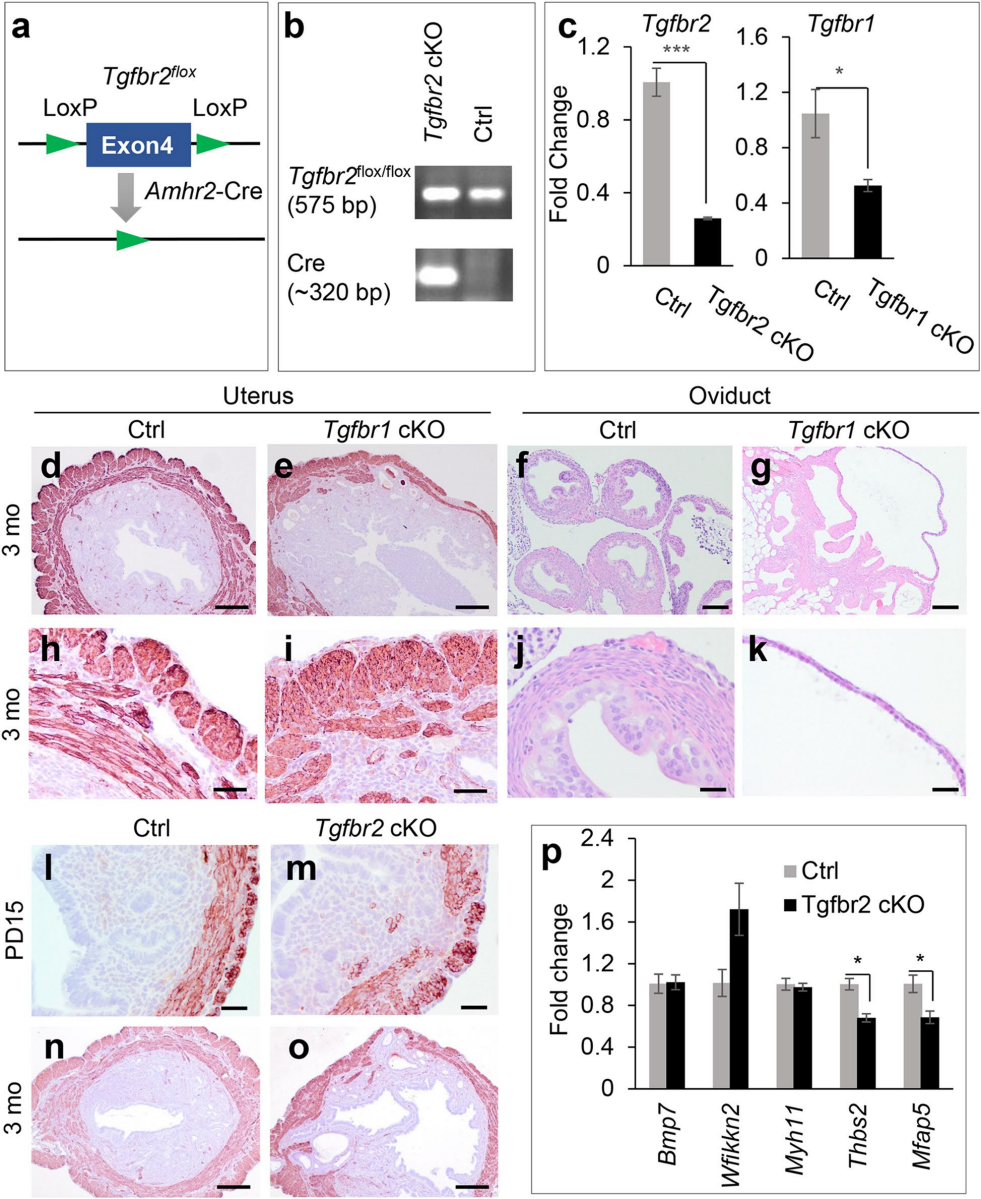
Loss of TGFBR2 disrupts myometrial formation. (**a**) Schematic representation of Cre-LoxP deletion of *Tgfbr2*. (**b**) Representative genotyping to identify *Tgfbr2* cKO and control mice. The full gel image is shown in Supplementary Fig. S1. (**c**) Reduced mRNA levels of *Tgfbr2* and *Tgfbr1* in the uteri of *Tgfbr2* cKO and *Tgfbr1* cKO at PD15, respectively. *n* = 3–4. *P < 0.05, ***P < 0.001. (**d–k**) Immunostaining of ACTA2 in the uteri and H.E. staining of oviducts using controls and *Tgfbr1* cKO at 3 months of age. (**h**–**k**) Represent higher magnification images for (**d**–**g**). (**l–o**) Immunostaining of ACTA2 in the uteri of controls and *Tgfbr2* cKO at PD15 and 3 months. Three independent mice were used for each genotype. (**p**) Transcript levels of *Bmp7, Wfikkn2, Myh11, Thbs2*, and *Mfap5* in the uteri of controls and *Tgfbr2* cKO at PD15. *n* = 3. *P < 0.05. Scale bar equals 25 µm (**j**,**k**,**l**,**m**), 50 µm (**h**,**i**), 100 µm (**f**,**g**), and 250 µm (**d**,**e**,**n**,**o**).

To determine the role of TGFBR2 in the female reproductive tract, we generated *Tgfbr2* conditional knockout (cKO) using *Amhr2*-Cre (Fig. 1a,b, Fig. S1). *Tgfbr1*^*flox/flox*^; *Amhr2*-Cre mice (*Tgfbr1* cKO) were examined to ensure that the reproductive phenotype upon *Tgfbr1* deletion can be replicated in the current experimental setting, where two *Tgfbr1*^flox^ alleles were used to generate mice with conditional deletion of *Tgfbr1*. In contrast, one floxed allele and one null allele of *Tgfbr1* (i.e., *Tgfbr1*^*flox/−*^; *Amhr2*-Cre) were used in our previous study^5^. The reduction of *Tgfbr2* and *Tgfbr1* mRNA levels was demonstrated in the uteri of *Tgfbr2* cKO and *Tgfbr1* cKO, respectively, compared with age-matched controls (Fig. 1c). Consistent with our previous report, we found that *Tgfbr1*^*flox/flox*^; *Amhr2*-Cre mice developed myometrial defects (Fig. 1e,i) and oviductal diverticula (Fig. 1g,k) compared with the respective uteri (Fig. 1d,h) and oviducts (Fig. 1f,j) of control mice. This result suggests that the reproductive phenotype of *Tgfbr1* cKO using *Amhr2*-Cre is independent on the number of *Tgfbr1* floxed alleles.

To test whether conditional deletion of *Tgfbr2* led to a similar myometrial abnormality to that of conditional deletion of *Tgfbr1*, we first performed immunostaining of smooth muscle actin alpha (ACTA2), a smooth muscle marker, using uteri from *Tgfbr2* cKO and controls at postnatal day 15 (PD15), when the basic configuration of the uterus is established^19^. Interestingly, disruption of the myometrial layers was found in *Tgfbr2* cKO but not in controls (Fig. 1l,m). Using uterine samples from 3-month-old *Tgfbr2* cKO and controls, we demonstrated that the observed myometrial defects persisted to the adulthood of *Tgfbr2* cKO (Fig. 1o). In contrast, the control uteri contained highly organized myometrial layers (Fig. 1n). This finding suggests an irreversible effect of TGFBR2 ablation on uterine smooth muscle development.

To explore potential molecular changes upon conditional deletion of *Tgfbr2*, we analyzed a number of genes expressed in distinct subcellular compartments of the endometrium and myometrium^20^. These candidate genes include bone morphogenetic protein 7 (*Bmp7*; inner stroma), WAP, follistatin/kazal, immunoglobulin, kunitz and netrin domain containing 2 (*Wfikkn2*; outer stroma), *Myh11* (inner myometrium), thrombospondin 2 (*Thbs2*; outer myometrium), and microfibril-associated protein 5 (*Mfap5*; interstitial myometrium)^20^. Quantitative reverse transcription-PCR (qRT-PCR) showed that *Thbs2* and *Mfap5* mRNA levels were decreased in the uteri of *Tgfbr2* cKO compared with controls (Fig. 1p). However, *Bmp7*, *Myh11*, and *Wfikkn2* transcript levels remained unchanged (Fig. 1p). The molecular changes specific to the outer and interstitial myometrium in *Tgfbr2* cKO indicate a reduction of gene expression in these cellular compartments or an alteration of cellular compositions of smooth muscle layers during early postnatal uterine development. Reduction of *Thbs2* mRNA levels was also found in the uteri of *Tgfbr1* cKO (Fig. S2).

**Figure 2:**
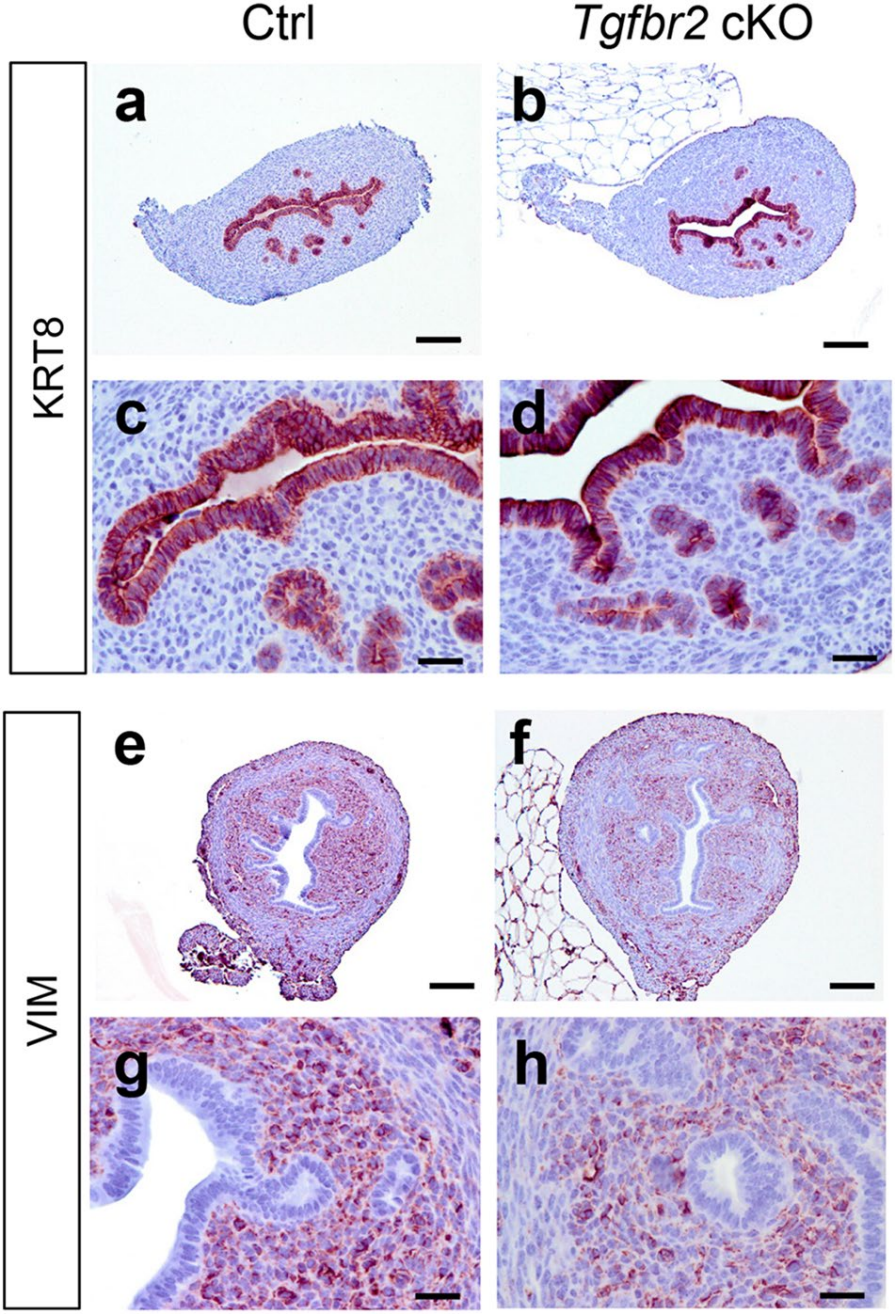
Normal specification of epithelial and stromal compartments in *Tgfbr2* cKO during early postnatal development. (**a–h**) Immunostaining of KRT8 and VIM in the uteri of controls and *Tgfbr2* cKO at PD15. (**c**,**d**,**g**,**h**) Are higher magnification images for (**a**,**b**,**e**,**f**), respectively. Three independent mice were examined for each genotype. Scale bar equals 25 µm (**c**,**d**,**g**,**h**) and 100 µm (**a**,**b**,**e**,**f**).

To further determine whether *Tgfbr2* deletion affected the formation of uterine epithelial and endometrial compartments, we performed cytokeratin 8 (KRT8) and vimentin (VIM) staining. KRT8 staining was found in both luminal and glandular epithelia (Fig. 2a–d), while VIM was localized to uterine stroma (Fig. 2e–h) of controls and *Tgfbr2* cKO at PD15. This evidence indicates normal specification and formation of the epithelial and stromal compartments in *Tgfbr2* cKO during early postnatal development.

### Formation of oviductal diverticula in *Tgfbr2* cKO

**Figure 3:**
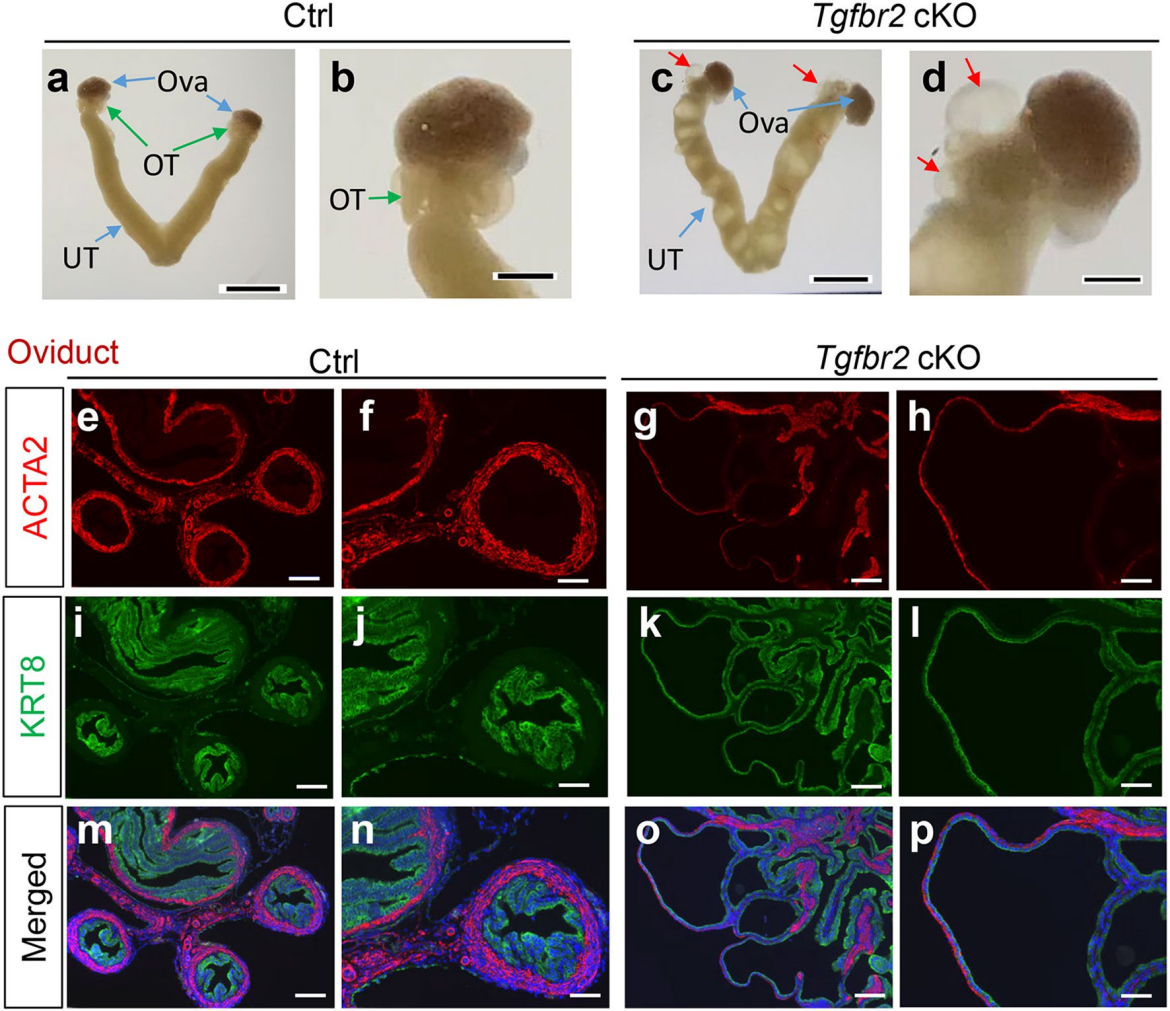
Formation of oviductal diverticula in *Tgfbr2* cKO. (**a**–**d**) Representative images of the reproductive tract from controls and *Tgfbr2* cKO at 3 months of age. UT, uterus; OT, oviduct; Ova, ovary. Red arrows in (**c**) and (**d**) indicate the diverticula. Scale bar = 4 mm (**a**,**c**) and 1 mm (**b**,**d**). (**e–p**) Double indirect immunofluorescence of ACTA2 and KRT8 staining using oviducts from controls and *Tgfbr2* cKO at 3 months of age. (**f**,**h**,**j**,**l**,**n**,**p**) Are higher magnification images for (**e**,**g**,**i**,**k**,**m**,**o**). Three independent mice were examined for each genotype. Scale bar equals 50 µm (**f**,**h**,**j**,**l**,**n**,**p**) and 100 µm (**e**,**g**,**i**,**k**,**m**,**o**).

We have previously shown that conditional deletion of *Tgfbr1* using *Amhr2*-Cre provokes oviductal abnormalities characterized by the formation of diverticula with impaired smooth muscle layers^5^. It was found that *Tgfbr2* cKO also developed oviductal diverticula (Fig. 3c,d), in stark contrast to controls (Fig. 3a,b). Immunofluorescence staining of ACTA2 and KRT8 revealed that the oviductal diverticulum from 3-month-old *Tgfbr2* cKO generally contained a single layer of flattened KRT8-positive epithelial cells and a weakened outer smooth muscle layer, in contrast to controls (Fig. 3e–p). A similar result was obtained using immunohistochemical analysis of oviducts from 2-month-old controls and *Tgfbr2* cKO (Fig. S3). In addition, ovaries from *Tgfbr2* cKO were immunostained with anti-ACTA2 antibody to better visualize the follicle structure. These ovaries appeared morphologically normal, containing follicles at various developmental stages (Fig. S4). These data indicate that TGFBR2, similar to its physiologic partner TGFBR1, is functionally required for the female reproductive tract development.

### Development of endometrial pathology in adult mice with *Tgfbr2* deletion

**Figure 4:**
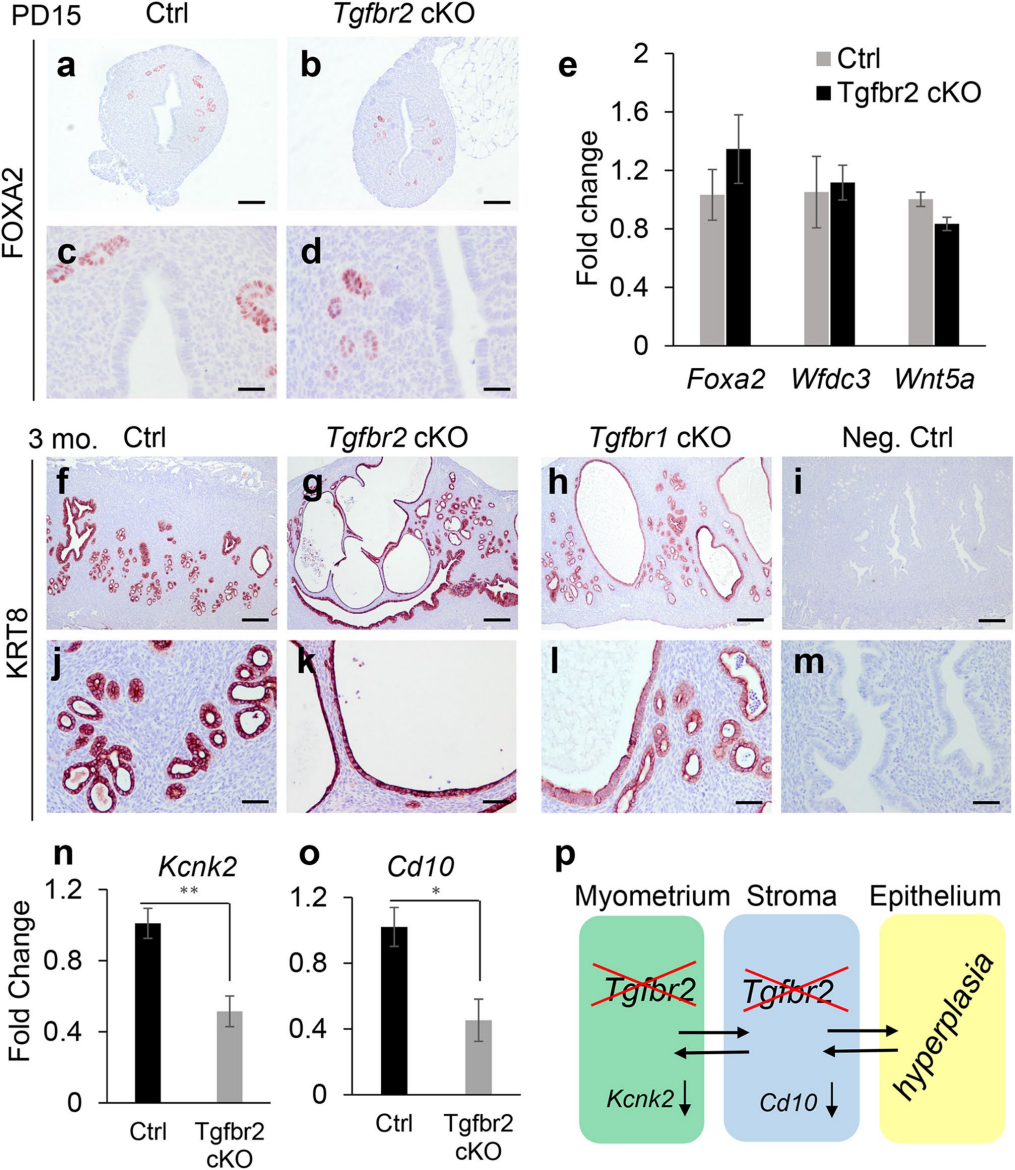
Development of endometrial pathology in adult *Tgfbr2* cKO. (**a–d**) Immunostaining of FOXA2 in the uteri of controls and *Tgfbr2* cKO at PD15. (**e**) Transcript levels of gland-associated genes in the uteri of controls and *Tgfbr2* cKO at PD15. *n* = 3. (**f–m**) Immunolocalization of KRT8 in the uteri of controls, *Tgfbr2* cKO, and *Tgfbr1* cKO. Negative controls using isotype-matched IgG are representatively shown in (**i**,**m**). (**c**,**d**,**j**–**m**) Represent higher magnification images for (**a**,**b**,**f**–**i**). Three independent mice were examined for each genotype. Scale bar equals 25 µm (**c**,**d**), 50 µm (**j**–**m**), 100 µm (**a**,**b**) and 250 µm (**f**–**i**). (**n**,**o**) Transcript levels of *Kcnk2* and *Cd10* in the uteri of controls and *Tgfbr2* cKO at PD10. *n* = 4. *P < 0.05, **P < 0.01. (**p**) Potential interactions among uterine smooth muscle cells, stromal cells, and epithelial cells.

Based on our previous finding that conditional deletion of *Tgfbr1* induces age-dependent glandular abnormalities and the development of hyperplastic endometrium^5,7^, we hypothesized that conditional ablation of TGFBR2 would lead to endometrial lesions as the pathological changes of the uterus progressed. To examine the morphological and molecular properties of uterine glands in *Tgfbr2* cKO, we performed immunostaining of uterine gland-specific marker forkhead box A2 (FOXA2)^21^ using uteri from controls and *Tgfbr2* cKO at PD15 and 3 months. At PD15, *Tgfbr2* cKO, similar to controls, contained morphologically normal uterine glands that were positively stained for FOXA2 (Fig. 4a–d), suggesting that TGFBR2 is dispensable for early postnatal adenogenesis*.* Consistent with the normal morphogenesis of uterine glands, our gene expression analysis showed that mRNA levels for uterine gland-specific genes including *Foxa2*, WAP four-disulfide core domain 3 (*Wfdc3*), and WNT family member 5A (*Wnt5a*) were comparable between *Tgfbr2* cKO and controls (Fig. 4e). However, at 3 months of age, cystic endometrial structures were observed in some *Tgfbr2* cKO (Fig. 4g,k), but not in age-matched controls (Fig. 4f,j). *Tgfbr1* cKO was included for the purpose of comparison. To better visualize the epithelial pathology, immunostaining was performed using longitudinal sections to minimize the potential regional bias of cross sections. Cystic gland-like structures were also identified in *Tgfbr1* cKO at the age of 3 months (Fig. 4h,l), supporting a similar role of TGFBR2 and TGFBR1 in the endometrial compartment. As evidence of epithelial cell proliferation, variable degrees of Ki67 staining were found in epithelial cells of cystic endometrial structures in *Tgfbr2* cKO (Fig. S5). Negative controls are depicted in Fig. 4i,m.

Because *Amhr2*-Cre activity is restricted to the myometrium and the stroma, the observed epithelial abnormalities were likely due to altered stromal-epithelial interactions. To determine whether disrupted smooth muscle formation affected myometrial function, we examined the expression of potassium channel subfamily K, member 2 (*Kcnk2*) encoding the two-pore domain potassium channel, which stabilizes membrane potential and maintains uterine quiescence during pregnancy^22^. We found that mRNA levels of *Kcnk2* were decreased in the uteri of *Tgfbr2* cKO at PD10 (Fig. 4n), indicating the altered ion channels and myometrial function upon conditional deletion of *Tgfbr2*. As disruption of stromal-myometrial interaction occurs in adenomyosis, a pathological condition characterized by the presence of uterine glands/stroma inside the smooth muscle compartment^23^, we speculated that the disorganized myometrium might impact endometrial differentiation. Supporting this idea, we found significantly reduced expression of membrane metallo endopeptidase (Mme/*Cd10*), a gene expressed in normal endometrial stroma^24^, in the uteri of *Tgfbr2* cKO at PD10 (Fig. 4o). This finding provides the developmental basis for potentially altered mesenchymal–epithelial interactions in the formation of hyperplastic endometrium in adult *Tgfbr2* cKO (Fig. 4p).

**Figure 5:**
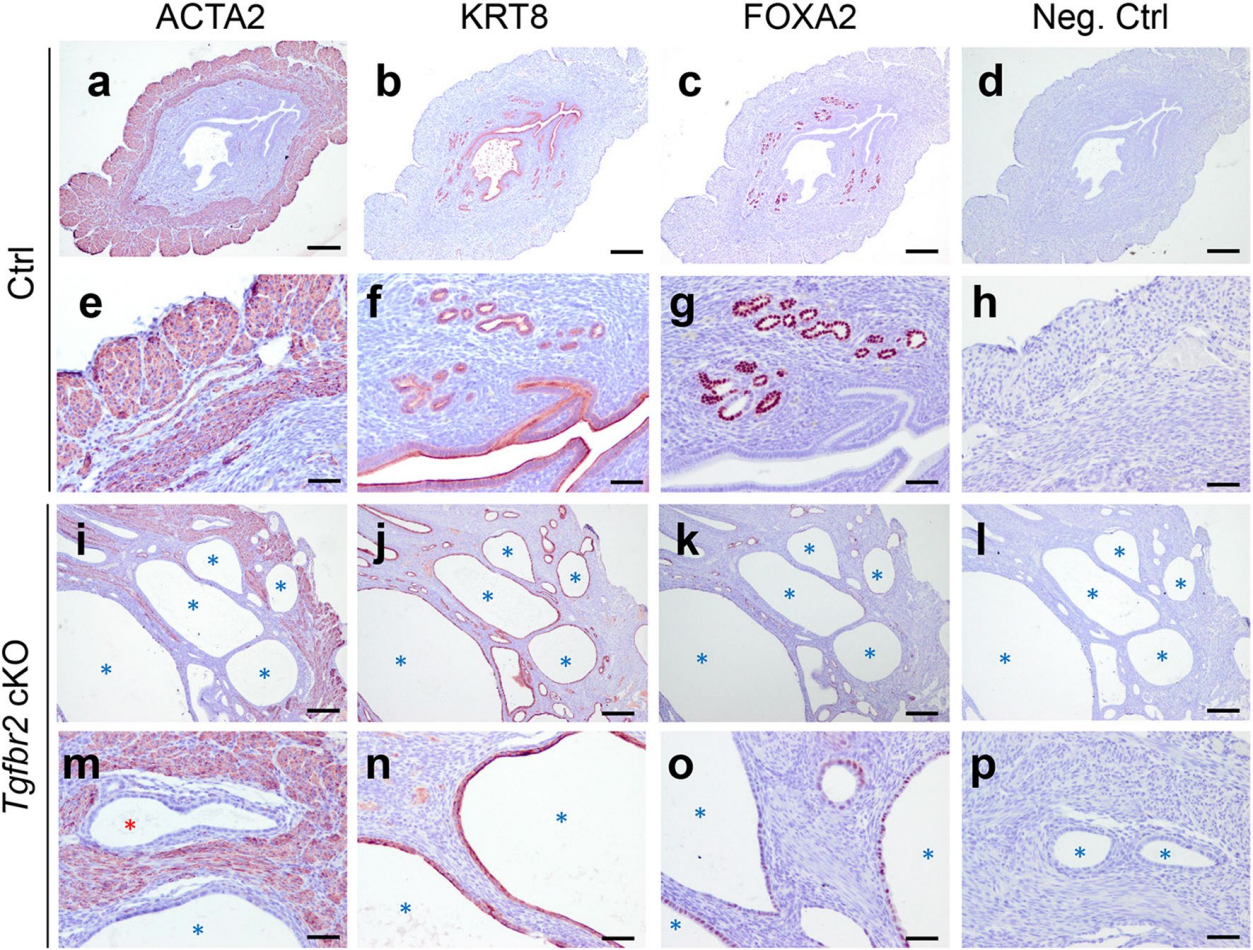
Formation of prominent cystic endometrium in *Tgfbr2* cKO at 6 months of age. (**a–p**) Immunohistochemical staining of ACTA2, KRT8, and FOXA2 using uteri from 6-month-old *Tgfbr2* cKO and controls. Representative negative controls using rabbit IgG are shown in (**d**,**h**,**l**,**p**). (**e**–**h** and **m**–**p**) Are higher magnification images for (**a**–**d** and **i**–**l**), respectively. Three independent mice were examined for each genotype. Scale bar equals 250 µm (**a**–**d** and **i**–**l**) and 50 µm (**e**–**h** and **m**–**p**). Cystic gland-like structures are indicated by asterisks.

In addition, we found that the endometrial pathology observed in *Tgfbr2* cKO was exacerbated with age. Mouse uteri were analyzed at the age of 6 months using immunostaining of ACTA2, KRT8, and FOXA2. Results showed that ACTA2-marked uterine smooth muscle layers were highly disorganized in *Tgfbr2* cKO (Fig. 5i,m), with the presence of prominent cystic structures positively stained for KRT8 in the endometrium (Fig. 5j,n). These findings were in sharp contrast to those of controls (Fig. 5a,b,e,f). Notably, some uterine epithelia in *Tgfbr2* cKO were mislocated to the myometrial compartment (Fig. 5m; red asterisk), as resembles adenomyosis previously found in mice with conditional deletion of *Tgfbr1*^5^. Uterine glands were labeled with FOXA2 (Fig. 5c,g) in controls. However, the cystic structures contained a mixed degree of FOXA2 staining (Fig. 5k,o). Negative controls showed minimal background staining of uterine samples from both controls and *Tgfbr2* cKO (Fig. 5d,h,l,p).

### Compound deletion of *Tgfbr2* and *Tgfbr1* phenocopies single deletion of each receptor

**Figure 6:**
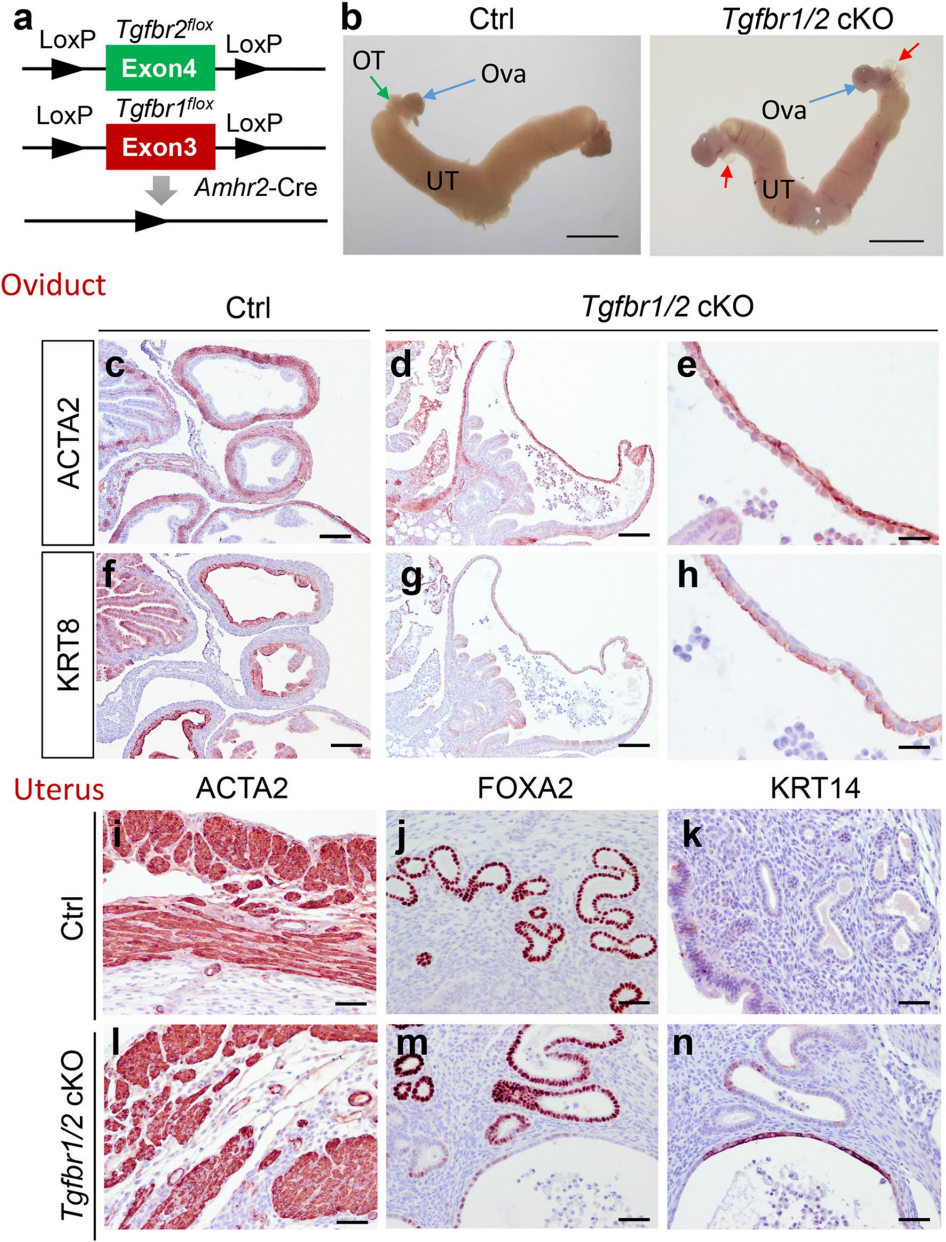
Phenotypic characterization of *Tgfbr1/2* cKO. (**a**) Schematic representation of generation of *Tgfbr1/2* cKO. (**b**) Gross image of uteri from controls and *Tgfbr1/2* cKO at 3 months of age. UT, uterus; OT, oviduct; Ova, ovary. Scale bar = 4 mm. (**c–h**) Localization of ACTA2 and KRT8 in the oviducts of controls and *Tgfbr1/2* cKO at 3 months of age. (**e**,**h**) Are higher magnification images for (**d**,**g**). (**i–n**) Immunostaining of ACTA2, FOXA2, and KRT14 using uteri from controls and *Tgfbr1/2* cKO at 3 months of age. Three independent mice were examined for each genotype. Scale bar equals 25 µm (**e**,**h**), 50 µm (**i**–**n**), 100 µm (**c**,**d**,**f**,**g**).

To further corroborate our finding that TGFBR2 and TGFBR1 played a similar role in the female reproductive tract, we generated mice that were conditionally deleted for both *Tgfbr2* and *Tgfbr1* (i.e., *Tgfbr1/2* cKO) (Fig. 6a) and performed phenotypic characterization. Results showed that conditional deletion of *Tgfbr1/2* resulted in a similar phenotype to individual deletion of *Tgfbr2* or *Tgfbr1*. *Tgfbr1/2* cKO developed oviductal diverticula under macroscopic analysis (Fig. 6b). Further immunohistochemical analysis revealed structural defects in the oviduct of *Tgfbr1/2* cKO. The oviductal diverticulum contained a layer of flattened epithelial cells marked by KRT8 staining and weakened smooth muscle walls visualized by ACTA2 staining (Fig. 6d,e,g,h), in contrast to controls (Fig. 6c,f). These mice also demonstrated myometrial abnormalities, evidenced by ACTA2 staining (Fig. 6l). Endometrial glands were labeled with FOXA2, with cystic gland-like structures found in some 3-month-old *Tgfbr1/2* cKO versus controls (Fig. 6m). Control mice showed well-organized myometrial structure and uterine glands (Fig. 6i,j).

**Figure 7:**
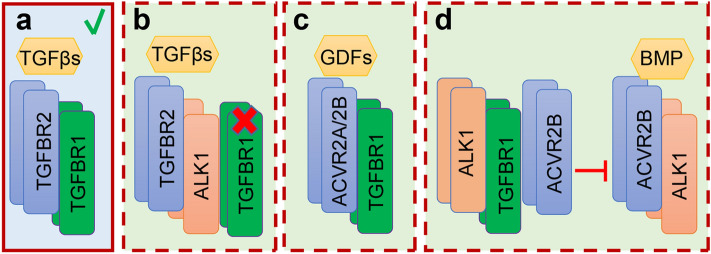
Contextually dependent TGFBR2/TGFBR1 signaling paradigms. (**a–d**) Potential modes of actions of TGFBR2 and TGFBR1 in different experimental systems. TGFBR2 is well known to complex with TGFBR1 to transduce signals by TGFβs (**a**). However, TGFBR2 may interact with other type 1 receptors (e.g., ALK1) in the absence of TGFBR1 to mediate TGFβ signaling that is detrimental to the homeostasis of aortic wall^15^ (**b**). On the other hand, TGFBR1 may bind to receptors besides TGFBR2 (e.g., ACVR2B) to mediate growth differentiation factor (GDF) signaling during craniofacial development^18^ (**c**). Moreover, TGFBR1 can interact with ALK1 and ACVR2B to suppress the formation of ACVR2B/ALK1 complex, thereby inhibiting BMP signaling^17^ (**d**). Our studies using conditional deletion of *Tgfbr2*, *Tgfbr1*, and *Tgfbr1/2* suggest the functional similarity between TGFBR2 and TGFBR1 in maintaining the developmental integrity of the female reproductive tract (**a**).

KRT14 is generally absent in normal uterine epithelial cells, but is expressed in basal cells of stratified epithelium^25^. Thus, expression of KRT14 is often associated with stratified epithelia^26^. To examine whether the development of cystic gland-like structures was associated with altered uterine epithelial identity, we performed immunostaining of KRT14 using uteri from *Tgfbr1/2* cKO and controls. Interestingly, although the epithelia of abnormal cystic structures morphologically resembled the simple epithelium, KRT14 staining was detectable in some of these structures (Fig. 6n), in contrast to controls where KRT14 was absent (Fig. 6k). Low magnification images for panels (i–n) are shown in Fig. S6. This result suggests that aberrant expression of KRT14 is associated with the impairment of uterine epithelial integrity. Results from *Tgfbr2* cKO, *Tgfbr1* cKO, and *Tgfbr1/2* cKO suggest the functional equivalence between TGFBR2 and TGFBR1 in maintaining the structural integrity of the female reproductive tract, in contrast to the signaling paradigm observed in several other systems (Fig. 7). Our findings, together with those from others^15–17^, further support the tissue/cell type-dependent functional similarity or divergence between these two receptors.

## Discussion

TGFβ signaling relies on the cell-surface TGFBR2/TGFBR1 complex^27^, where TGFBR2 activates TGFBR1 prior to the initiation of SMAD-associated signal transduction^2^. In the current study, we showed that conditional depletion of TGFBR2 led to a similar phenotype in the female reproductive tract as ablation of TGFBR1. The structural defects resulting from deletion of either receptor were manifested by the development of oviductal diverticula, disorganized myometrial layers, and endometrial abnormalities. Results support a similar role of TGFBR2 and TGFBR1 in maintaining the integrity of the female reproductive tract.

The proper differentiation of the female reproductive tract is critical for successful pregnancy^28^. Our loss-of-function and gain-of-function studies of *Tgfbr1* suggest that balanced TGFβ signaling is required for the development of the myometrium and the uterine gland^5–7,29^. While depletion of TGFBR1 using *Amhr2*-Cre that is only expressed in the mesenchymal compartment of the uterus causes myometrial defects^5–7^, deletion of *Tgfbr1* in the uterine epithelium, stroma, and smooth muscle using progesterone receptor (*Pgr*)*-*Cre results in abnormal placental development and metastatic endometrial cancer, which appears to be contingent upon a pregnancy-associated remodeling event of the endometrium^30,31^. The absence of myometrial abnormality in *Tgfbr1 Pgr*-Cre cKO suggests an important role of TGFBR1 in early postnatal reproductive tract development^32^. Collectively, these findings indicate that TGFBR1 is a key regulator of the female reproductive tract development, pregnancy, and endometrial carcinogenesis. While the role of TGFBR1 has been extensively investigated in reproduction, the function of TGFBR2 remains largely unknown.

To elucidate the function of TGFBR2 in the female reproductive tract, we created *Tgfbr2* cKO in the current study, and found similar myometrial disruption and oviductal abnormalities in both *Tgfbr2* cKO and *Tgfbr1* cKO. Moreover, genetic removal of *Tgfbr1* in the *Tgfbr2* conditional knockout background showed minimal impact on the reproductive tract phenotype of *Tgfbr2* cKO. It was interesting to note that some cystic gland-like structures in *Tgfbr1/2* cKO expressed KRT14. As KRT14 is rarely expressed in the single-layer epithelium, its detection in these cells indicates impaired epithelial integrity. However, the phenotype of endometrial abnormality is not specific to *Tgfbr1/Tgfbr2* double deletion, because some cystic structures in *Tgfbr2* cKO or *Tgfbr1* cKO were also stained for KRT14 (unpublished observation). As *Amhr2*-Cre is not expressed in uterine epithelial cells^33,34^, the effect of *Tgfbr2/Tgfbr1* deletion on the endometrial abnormalities was likely associated with altered stromal-epithelial cell interactions. This concept is supported by findings from the Cunha laboratory demonstrating that organ-specific stromal factors are critically important for the differentiation of uterine and vaginal epithelia using tissue recombinant approach^35^.

Our findings that defective myometrial and oviductal formation was manifested upon *Tgfbr2* or *Tgfbr1* deletion and that loss of TGFBR1 did not alter the phenotype induced by *Tgfbr2* deletion suggest that the signaling axis of TGFβ ligand(s) → TGFBR2 → TGFBR1 is critically important for maintaining the integrity of the female reproductive tract. Unlike observations of different phenotypic consequences resulting from loss-of-function of TGFBR1 versus TGFBR2 in several model systems^15–17^, our results indicate the functional similarity between TGFβ type 1 and type 2 receptors in the female reproductive tract. While our findings and the aforementioned reports^15–17^ collectively suggest tissue-dependent TGFBR1/TGFBR2 signaling paradigms, mechanisms governing the tissue type-specific roles of and functional interactions between TGFBR2 and TGFBR1 are unclear. It is plausible that the differences are related to compensatory pathways developed or available functional signaling components expressed in a given tissue upon the inactivation of one of the receptors.

Canonical TGFβ signal transduction depends on SMAD proteins; however, the downstream SMAD signaling that mediates the oviductal and uterine phenotype remains elusive. Of note, the oviductal and myometrial phenotypes have not been reported in *Smad2/3* conditional knockout mice^36^. Rodriguez et al. has analyzed the reproductive tract phenotype of five independent mouse lines with conditional deletion of *Smad1*, *Smad5*, *Smad1/5*, *Smad4*, and *Smad1/5/4* using *Amhr2*-Cre recombinase^37^. It was found that *Smad1/5/4* cKO, but not the others, develop oviductal and myometrial defects^37^. We have previously reported that the development of oviductal diverticula in *Tgfbr1* cKO mice prevents embryos from entering the uterus; no embryos can be retrieved from the uterus of *Tgfbr1* cKO^5^. It appears that the oviductal phenotype of *Smad1/5/4* cKO is less severe than that of *Tgfbr1* cKO, since embryos, even though with reduced numbers, can still be recovered from the uterus of *Smad1/5/4* cKO at E4.5^37^. As further support of the less severe oviductal phenotype in *Smad1/5/4* cKO, the development of oviductal diverticula is not apparent by gross examination prior to 6 weeks of age^37^. In contrast, an apparent oviductal phenotype is visible in *Tgfbr1* cKO at 3 weeks of age^5^. TGFβ signaling is known to activate both TGFβ- and BMP-associated SMADs^38^; and, therefore, it is conceivable that both SMAD2/3 and SMAD1/5/9 are implicated in this developmental process. Of note, conditional ablation of SMAD4 per se, the common SMAD that mediates both TGFβ and BMP signaling, is insufficient to induce this phenotype. Thus, it is tempting to speculate that SMAD1/5-dependent but SMAD4-independent signaling is involved in maintaining the structural integrity of the reproductive tract. Indeed, such atypical BMP signaling has been demonstrated in early odontogenesis^39^. In addition, it has been shown that SMAD2/3-dependent, but SMAD4-independent, signaling is involved in microRNA processing^40^. Further research efforts are warranted to understand how SMAD signaling safeguards the reproductive tract integrity and how its dysregulation contributes to reproductive abnormalities and disease.

In summary, we found that conditional deletion of *Tgfbr2* or *Tgfbr1* resulted in a similar phenotype characterized by oviductal diverticulum formation and myometrial disruption, lending support to the functional requirement of these receptors in the female reproductive tract. This study also expanded our genetic tool kit that can be utilized to understand the pathogenesis of oviduct-, myometrium-, and uterine epithelium-associated disorders and diseases that adversely affect pregnancy outcome and fertility. The *Tgfbr2* cKO may serve as a valuable reference model in dissecting the contextually dependent function of TGFBR2 and TGFBR1, in terms of the development of compensatory signaling branches and/or interactions between one receptor with available signaling components upon inactivation of the other.

## Materials and methods

### Study approval

All experiments involving live animals were conducted in compliance with the guidelines and regulations for animal use and care by the National Institute of Health. All procedures of mouse handling and use were approved by the Institutional Animal Care and Use Committee (IACUC) at Texas A&M University (protocol number: 2018-0005). Every effort has been made to minimize discomfort and pain during experimentation. The reporting of experiments was in compliance with the ARRIVE guidelines.

### Animals

Mice were maintained on a mixed C57/BL6/129SvEv background. Sample size for each experiment was indicated in the figure legends, and was based on our previous observation of the phenotypic variation in *Tgfbr1* mutant mice^5^. No animals were excluded from the experiment. No randomization was utilized, as there was no treatment of mice performed in this study. Researchers were not blinded to the group allocation. Generation of *Tgfbr1* floxed mice is described elsewhere^41^. Mice containing *Tgfbr2* floxed alleles were obtained from the Jackson Laboratory (Stock No. 012603). The *Tgfbr1*^*flox/flox*^ mice and *Tgfbr2*^*flox/flox*^ mice were crossed with *Amhr2*-Cre mice to generate mice with *Tgfbr1*^*flox/flox*^*; Amhr2*-Cre (*Tgfbr1* cKO), *Tgfbr2*^*flox/flox*^*; Amhr2*-Cre (*Tgfbr2* cKO), and *Tgfbr1*^*flox/flox*^*; Tgfbr2*^*flox/flox*^*; Amhr2*-Cre (*Tgfbr1/2* cKO). Genomic PCR was conducted to analyze the genotypes of mice using tail DNA^5,42^ and gene-specific primers for *Tgfbr1*^flox^ ^5^, *Tgfbr2*^flox^ [5′-TATGGACTGGCTGCTTTTGTATTC-3′ and 5′- TGGGGATAGAGGTAGAAAGACATA-3′ (wild-type band = 422 bp and flox band = 575 bp)]^5,42^, and *Amhr2*-Cre^5^. Expression of *Tgfbr1* and *Tgfbr2* mRNA was determined using qRT-PCR and primers for*Tgfbr1*^5^ and *Tgfbr2* (5′-GACCACACTCCTTGTGGGAG-3′ and 5′-AGGCAACAGGTCAAGTCGTT-3′).

### Tissue collection and preparation

Uterine, ovarian, and oviductal tissues were collected from *Tgfbr1* cKO, *Tgfbr2* cKO, and *Tgfbr1/2* cKO and corresponding controls at timepoints indicated in the results section. At least three mice were used for each genotype per timepoint. Tissues were fixed in 10% neutral buffered formalin, washed with 70% ethanol, embedded in paraffin, and serially sectioned for histology, immunohistochemistry, and immunofluorescence described below. Tissue processing was completed using the College of Veterinary Histology Research Laboratory at Texas A&M University.

### Histology and immunohistochemistry

Histological analysis was performed using paraffin-embedded sections (5 μm) and hematoxylin and eosin (H.E.) staining. Immunohistochemistry was performed to determine the localization of specific antigens in the reproductive tract as described previously^6^. The staining was performed using VECTASTAIN Elite ABC-HRP Kit (PK-6100; Vector Laboratories) according to the manufacturer’s instruction. In brief, sections were deparaffinized in xylene and rehydrated in graded alcohol, followed by antigen retrieval using citrate buffer (pH = 6). Slides were incubated with primary antibodies including rabbit anti-FOXA2 IgG (1:200; ab108422; Abcam), rat anti-KRT8 IgG (1: 200; TROMA-I; Developmental Studies Hybridoma Bank), rabbit anti-VIM IgG (1:200; #5741; Cell Signaling Technology), rabbit anti-Ki67 IgG (1:500; #12202; Cell Signaling Technology), rabbit anti-ACTA2 IgG (1:500; #19245; Cell Signaling Technology), and rabbit anti-KRT14 IgG (1:400; PA5-16722; Thermo Fisher Scientific) at 4 °C overnight. Incubation with biotinylated secondary anti-rabbit (BA-1000; Vector Laboratories) or anti-rat (BA-9400; Vector Laboratories) antibodies was conducted at room temperature. The NovaRED™ Peroxidase Substrate Kit (SK-4800; Vector Laboratories) was utilized for signal development. Sections were counterstained with hematoxylin and mounted using Permount media (Fisher Scientific). Results were examined under Olympus BX47 microscope and images captured using DP25 or LC30 camera.

### Indirect immunofluorescence

Indirect immunofluorescence was performed to visualize alterations of the smooth muscle and epithelium of the oviduct in mice with conditional deletion of *Tgfbr2*, *Tgfbr1*, or *Tgfbr1/2* as described^43^. In brief, paraffin-embedded sections were deparaffinized using xylene and rehydrated before antigen retrieval. After blocking, sections were incubated with primary antibodies including rabbit anti-ACTA2 IgG (1:500) and rat anti-KRT8 IgG (1: 200) at 4 °C overnight. Alexa Fluor 488- or 594-conjugated secondary antibodies were purchased from Invitrogen. After the completion of antibody incubation, mounting media containing 4ʹ,6-diamidino-2-phenylindole (DAPI) were applied to the slides. An Olympus IX73 microscope equipped with XM10 CCD camera was used to examine fluorescence signals and capture images via cellSens Software.

### qRT-PCR

Total RNA isolation and qRT-PCR were performed as previously reported^44^. Approximately 500 ng of total RNA per reaction was used for superscript III-based reverse transcription. The qRT-PCR reaction system contains cDNA, gene-specific primers, and iTaq Universal SYBR Green master mix (Bio-Rad) or Taqman Universal PCR Master Mix (Invitrogen). Relative gene expression was determined^45^, using ribosomal protein L19 (*Rpl19*) as an internal control^46^. Primers used for qRT-PCR include *Kcnk2* (5′-CCGAGGCTCTCATTCTCCTCA-3′ and 5′- AGGACGACCACCAGGAAAATC-3′; PrimerBank ID 6754432a1)^47^, *Cd10*^48^, *Bmp7* (5′-ACGGACAGGGCTTCTCCTAC-3′ and 5′-ATGGTGGTATCGAGGGTGGAA-3′; PrimerBank ID 31982487a1)^47^, *Myh11*^5^, *Wfikkn2* (Mm00725281_m1; Thermo Fisher Scientific), *Thbs2*^20^, *Mfap5* (Mm00489404_m1; Thermo Fisher Scientific)*, Foxa2*^48^*, Wfdc3*^48^, and *Wnt5a*^48^. At least three biological replicates were analyzed for each genotype, with two technical replicates included for each sample.

### Statistical analysis

Comparison of the difference between two groups was performed using two-tailed *t*-test with Microsoft Excel. Data are shown as mean ± s.e.m. Statistical significance was defined at *P* < 0.05, with results indicated as *P < 0.05, **P < 0.01, and ***P < 0.001.

## Supplementary Information

Supplementary Information.

## Data Availability

All data are included in this manuscript and its supplementary materials.
