# Supplementary material for: Functional similarity between TGF-beta type 2 and type 1 receptors in the female reproductive tract
Source: Sci Rep. 2021 Apr 29;11:9294. doi: 10.1038/s41598-021-88673-y (PMC8084965; doi:10.1038/s41598-021-88673-y)
Supplement: Supplementary file 1 — Supplementary Information. [file 41598_2021_88673_MOESM1_ESM.pdf]

Supplementary Materials

Functional Similarity between TGF-beta Type 2 and Type 1 Receptors in the Female Reproductive Tract

Nan Ni[1], Xin Fang[1], Qinglei Li[1,*]

[1]Department of Veterinary Integrative Biosciences, Texas A&M University, College Station, TX 77843, USA

***Correspondence:** Qinglei Li, Ph.D., Department of Veterinary Integrative Biosciences, College of Veterinary Medicine & Biomedical Sciences, Texas A&M University, College Station, Texas, 77843. Phone: 979 862 2009; Fax: 979 847 8981; E-mail: qli@cvm.tamu.edu

Table of contents:

Supplementary Figure S1

Supplementary Figure S2

Supplementary Figure S3

Supplementary Figure S4

Supplementary Figure S5

Supplementary Figure S6

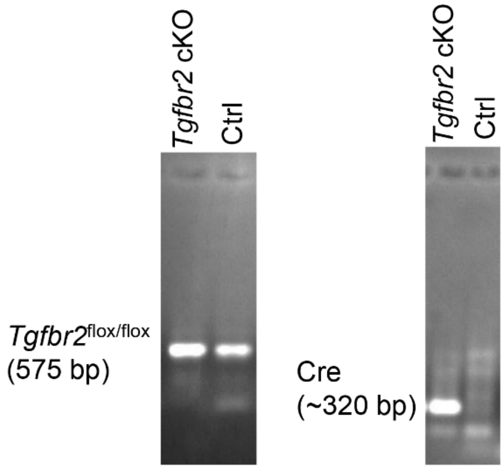

Figure S1. Full gel images for Fig. 1b.

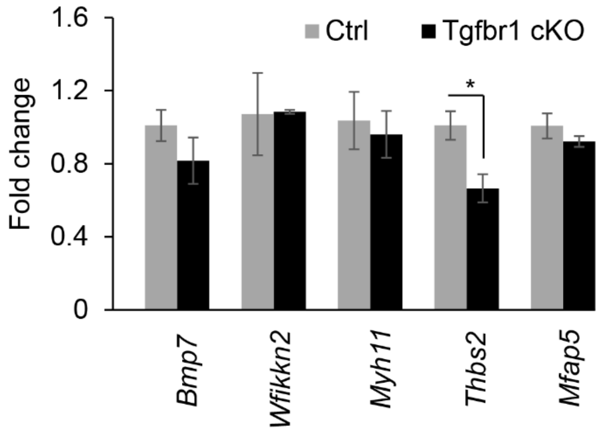

Figure S2. Transcript levels of *Bmp7, Wfikkn2, Myh11, Thbs2*, and *Mfap5* in the uteri of controls and *Tgfbr1* cKO at PD15. $n = 4$. $^{*}P < 0.05$.

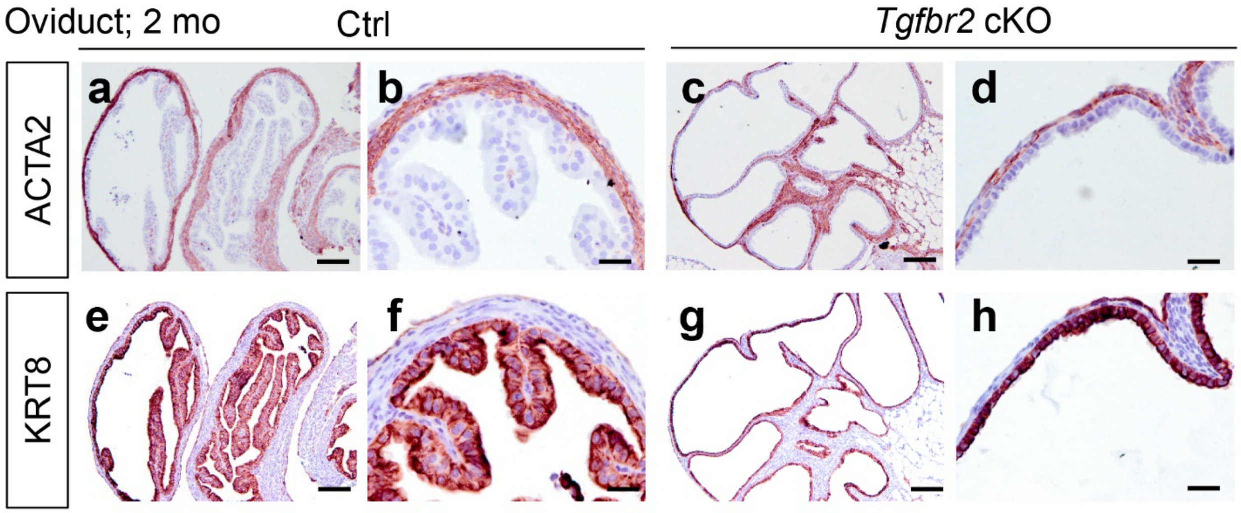

Figure S3. Development of oviductal diverticula in *Tgfbr2* cKO. (**a**-**h**) Immunostaining of ACTA2 and KRT8 using oviducts from controls and *Tgfbr2* cKO at 2 months of age. *n* = 3. Panels (b, d, f, and h) are higher magnification images for panels (a, c, e, and g). Scale bar equals 25 μm (b, d, f, and h) and 100 μm (a, c, e, and g).

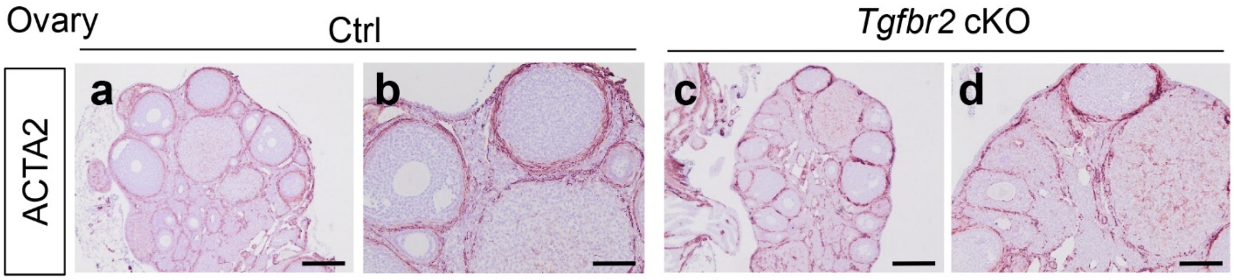

Figure S4. Morphological analysis of ovaries from *Tgfbr2* cKO. (**a**-**d**) immunostaining of ACTA2 using ovaries from controls and *Tgfbr2* cKO at 3 months of age. Panels (b and d) are higher magnification images for panels (a and c). Scale bar equals 100 μm (b and d) and 250 μm (a and c).

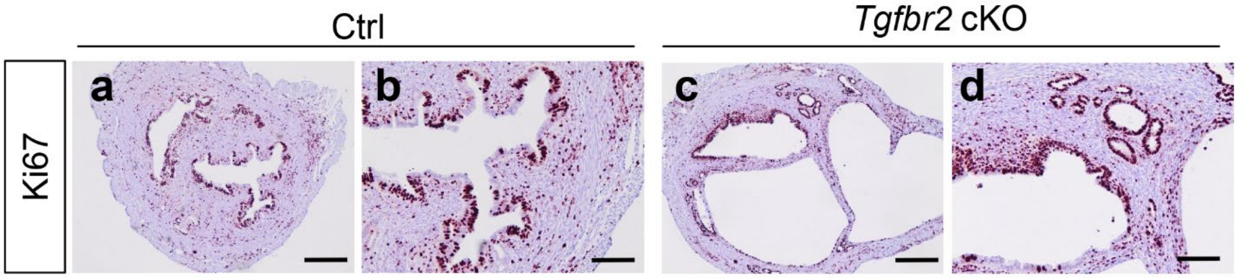

Figure S5. Immunostaining of Ki67 using uteri from *Tgfbr2* cKO at 3 months of age. Panels (b and d) are higher magnification images for panels (a and c). Scale bar equals 100 µm (b and d) and 250 µm (a and c).

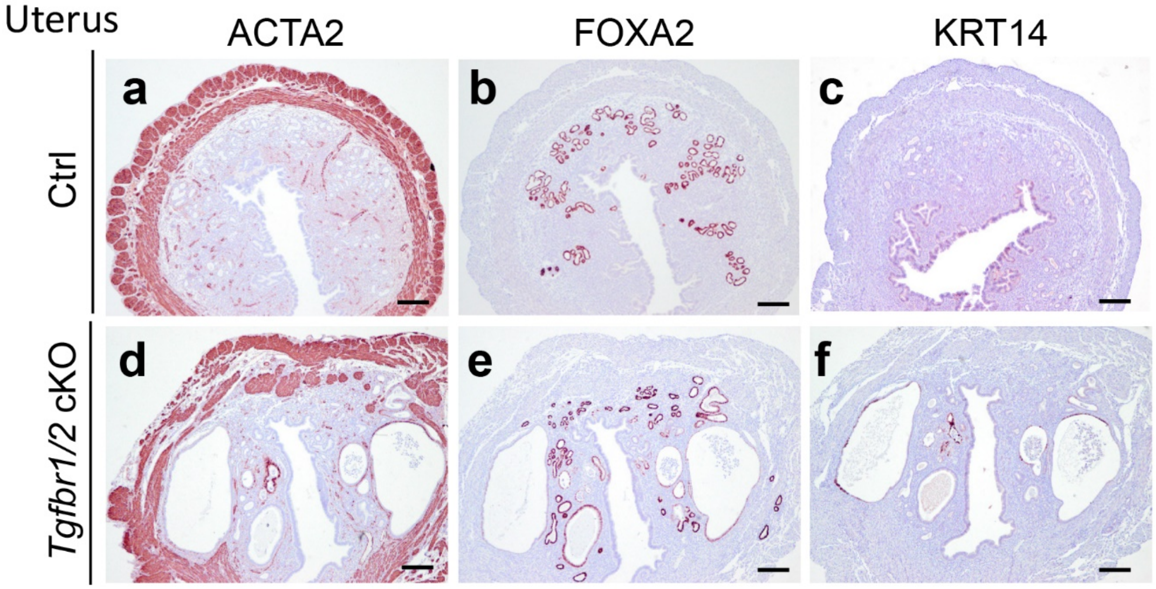

Figure S6. Low magnification images for uterine immunostaining of ACTA2, FOXA2, and KRT14 shown in Figure 6. Panels (**a**-**f**) represent low magnification images shown in panels (i-n) in Figure 6. Scale bar is representatively shown in (a) and equals 200 µm (a-f).
